# A risk model based on miR-483-5p and miR-150 for atrial fibrillation recurrence

**DOI:** 10.1007/s13205-026-04842-8

**Published:** 2026-05-20

**Authors:** Wenwen Lai, Hong Chen, Mingwei Huang, Zhendong Cheng, Naping Lin, Huiyao Lu

**Affiliations:** Department of Cardiovascular Medicine, Second Affiliated Hospital of Fujian Medical University, Quanzhou, 362000 China

**Keywords:** Atrial fibrillation, Radiofrequency ablation, MicroRNA, Computational mining, Risk stratification model, Predictive engineering

## Abstract

Atrial Fibrillation (AF) is a common arrhythmia with high recurrence after Radiofrequency Ablation, and the absence of universal predictive tools impedes clinical prognosis evaluation and treatment optimization. To address this gap, we developed a retrospective microRNA (miRNA)-based recurrence risk stratification model for post-RFA AF patients using an integrated bioinformatics and clinical validation approach. First, we performed differential expression analysis of two Gene Expression Omnibus (GEO) datasets (GSE144384, GSE71963) via GEO2R and R software (version 4.2.1, Limma package) with strict thresholds (|logFC| > 1.5, *P* < 0.05) identified upregulated miR-483-5p and downregulated miR-150 as core biomarkers. This finding was cross-validated across both datasets (*P* < 0.001). Subsequently, we enrolled 122 AF patients after RFA and 60 patients with sinus rhythm as controls, and we measured plasma miRNA levels by Quantitative real-time Polymerase Chain Reaction and left atrial functional/structural parameters via color Doppler ultrasound. Receiver Operating Characteristic curve analysis determined optimal cut-off values (miR-483-5p: 1.025; miR-150: 0.805) for constructing a three-tier recurrence risk stratification model. Validation during 12-month follow-up confirmed the model, showing gradient recurrence rates of 69.05%, 24.14% and 4.55% across high/medium/low-risk groups (*P* < 0.001). The combined Area Under the Curve for predictive performance was 0.888 (superior to that of single biomarkers). We propose a reproducible miRNA-driven predictive framework integrating public omics mining and clinical validation, providing an engineering-based tool for early identification of high-risk post-RFA patients and guidance for individualized treatment. The framework is extendable to other arrhythmias, laying a foundation for translational research of miRNA-based predictive models.

**Supplementary Information:**

The online version contains supplementary material available at 10.1007/s13205-026-04842-8.

## Introduction

In clinical practice, Atrial Fibrillation (AF) is a common arrhythmia that impairs cardiac pumping function and promotes blood stasis, leading to complications such as myocardial ischemia, thromboembolism, and heart failure (Hess et al. 2020; Qiu et al. 2019). Globally, 33.5 million individuals were affected by AF in 2010, and its prevalence has increased markedly with age, reaching approximately 12% in those aged 75 to 84 years and over 33% in individuals aged ≥ 80 years (Wang et al. 2021). In China, AF prevalence ranges from 0.49% to 8.8% in community populations and from 4.4% to 35.7% in hospitalized patients, imposing an increasing healthcare burden (Song 2022). Radiofrequency Ablation (RFA) is a first-line treatment for AF, which restores sinus rhythm by pulmonary vein isolation and ablating abnormal atrial tissue (Romero et al. 2021). Although the success rate of RFA reaches 89% in paroxysmal AF, outcomes in persistent AF remain suboptimal, with high recurrence rates and frequent need for repeat procedures (Njoku et al. 2018). Therefore, identifying reliable predictors of post-RFA recurrence is critical for treatment planning, patient selection, and prognosis improvement, and inflammatory markers such as high-sensitivity C-reactive protein have attracted increasing attention as potential predictors (Jaroonpipatkul et al. 2023).

MicroRNAs (miRNAs) are endogenous non-coding RNAs widely distributed in human tissues, which are involved in key cellular processes including cell differentiation, proliferation, and apoptosis (Xie et al. 2023). Accumulating evidence indicates that multiple miRNAs participate in atrial structural and electrical remodeling and contribute to the initiation and progression of AF (Costa et al. 2019; Li et al. 2019a). For example, miR-21 is significantly upregulated in the atrial tissues of AF models induced by myocardial infarction. By targeting the gene Sprouty-1, miR-21 knockdown can effectively reduce atrial fibrosis and inhibit AF progression (Cardin et al. 2012). In addition, miR-1 deficiency disrupts cardiac electrophysiological homeostasis, promotes atrial arrhythmogenicity, and is one of the main etiological factors of AF-related cardiac remodeling (Yang et al. 2024). However, most studies focus on individual miRNAs, and the complex regulatory networks of miRNAs in AF have not been fully elucidated, which creates gaps in the translation of basic research into clinical applications (Balan and Scridon 2025). Public databases including the Gene Expression Omnibus (GEO) provide microRNA (miRNA) expression profiles of AF patients, enabling the screening of AF-related miRNAs and supporting clinical validation. Building on prior research on miRNAs in AF, this study aimed to identify key miRNAs, clarify their roles in AF and predict post-RFA recurrence.

Machine learning has become an effective approach for identifying disease-related miRNAs and predicting disease outcomes from high-dimensional data (Quynh et al. 2025). Recent advances in deep learning have demonstrated its strong capability in modeling complex biological sequence-function relationships, highlighting its broad applicability in genomics and transcriptomics research (Wang et al. 2025). In AF research, machine learning models including random forest and support vector machine have been applied to Electrocardiogram-based (ECG-based) diagnosis with favorable accuracy (Sraitih et al. 2021). Beyond AF, machine learning has facilitated the development of miRNA signatures for disease prediction, such as a 3-miRNA panel (miR-138-5p, miR-146b-5p, miR-200a-3p) with 94% accuracy in distinguishing bladder cancer subtypes (Eckhart et al. 2025) and a random forest model for predicting COVID-19 severity using miRNA expression profiles (Ren et al. 2022). For neurodegenerative diseases like Alzheimer’s, integrating miRNAs into machine learning models significantly improved the classification of pathological subtypes, highlighting the versatility of this approach (Asanomi et al. 2021). In AF, several studies have explored miRNA-related recurrence prediction, and have identified markers such as miR-320a and miR-29b-3p (Zhan et al. 2024). However, current studies largely focus on single miRNAs and lack integrated validation combining public databases with clinical samples, as well as comprehensive analysis of miRNA associations with AF clinical characteristics and quantitative recurrence risk stratification.

Therefore, this study adopted a design combining bioinformatics analysis and clinical validation to systematically explore the roles of miR-483-5p and miR-150 in AF. First, relevant miRNA expression datasets were retrieved from the GEO database, with their correlations with AF verified via preprocessing and differential analysis. Subsequently, AF patients undergoing RFA in our hospital were enrolled as the study group and sinus rhythm patients as the control group, with clinical baseline data collected. Quantitative real-time polymerase chain reaction (qPCR) was used to detect plasma levels of the two miRNAs, and color Doppler ultrasound was used to assess left atrial function and structural parameters, exploring their associations with left atrial function and remodeling. After RFA, a long-term follow-up was conducted to monitor recurrence, and a practical miRNA-based predictive system for AF recurrence was established based on the two miRNAs’ expression to quantify the post-RFA recurrence risk. This study integrated database mining, clinical detection, functional correlation analysis, and prognostic stratification, aiming to provide a systematic reference for the post-RFA prognosis assessment and individualized treatment optimization in AF patients.

## Materials and methods

### General information

From April 2021 to October 2022, 122 AF patients (*n* = 122, representing independent clinical samples) who underwent RFA in our hospital were selected as the study group. Additionally, 60 patients with sinus rhythm (*n* = 60, independent clinical samples) during the same period were selected as the control group from the same hospital. This study was approved by the Ethics Committee of the Second Affiliated Hospital of Fujian Medical University (No. 2021YB1035). Inclusion criteria: (1) Patients who met the relevant diagnostic criteria of AF, confirmed by Holter and 12-lead ECG (January et al. 2019); (2) Patients received RFA; (3) Patients who were confirmed by Holter or body surface ECG that P-waves disappeared after surgery and were replaced by AF waves; (4) Patients or their family members signed the informed consent. Exclusion criteria: (1) Patients with a previous history of cardiac surgery or RFA; (2) Patients combined with rheumatic valvular heart disease, congenital heart disease, cardiomyopathy, etc.; (3) Patients with abnormal thyroid, kidney and liver function; (4) Patients with AF caused by cerebrovascular diseases such as cerebral infarction or cerebral hemorrhage; (5) Patients complicated with uncontrolled acute or chronic infectious diseases; (6) Patients combined with autoimmune diseases, malignant tumors or other severe diseases.

### Bioinformatics analysis of GEO database

First, datasets related to AF and miRNA expression profiles were retrieved from the GEO database, with the following inclusion criteria: (1) Study subjects were divided into an atrial fibrillation group and a sinus rhythm control group; (2) Sample types were plasma or serum; (3) Raw miRNA expression data were provided; (4) The total sample size was no less than 50 cases (with no fewer than 30 cases in the AF group and 20 cases in the control group) (Wei et al. 2022). Finally, two datasets were screened out: GSE144384 (*n* = 62 AF samples, *n* = 30 control samples; independent samples) and GSE71963 (*n* = 45 AF samples, *n* = 25 control samples; independent samples) Subsequently, data processing was performed using the GEO2R online tool and R software (version 4.2.1, Limma package). Raw expression data were processed with the Limma package. Background correction and quantile normalization were performed using the normalizeBetweenArrays function, and batch effects between datasets were corrected using the removeBatchEffect function. Differentially expressed microRNAs (DEmiRNAs) between the AF group and the control group were screened using the thresholds of |log Fold Change (logFC)| > 1.5 and *P* < 0.05. Meanwhile, the expression levels of miR-483-5p and miR-150 were extracted from the two datasets, and the inter-group differences were compared using the independent-samples t-test.

### Detection of miR-483-5p and miR-150

Within 24 h after admission, 3 mL of fasting venous blood (8–12 h fasting) was collected from each patient, placed in an anticoagulation tube, centrifuged (1500 r/min) for 15 min, and the plasma was separated. Plasma separation was completed within 1 h after centrifugation to avoid RNA degradation. Total RNA was extracted according to the instructions of the kit (No. 217184, QIAGEN, Germany, Batch No.128056729). After extraction, RNA samples were immediately stored at −80 °C and reverse transcription was performed within 7 days to minimize RNA degradation. The RNA was reverse-transcribed into cDNA using the MiScript II RT Kit (No. 218161, QIAGEN, Germany, Batch No: 129045813). The levels of miR-483-5p and miR-150 were detected by fluorescence quantitative PCR with the MiScript SYBR Green Kit (No. 218073, QIAGEN, Germany, Batch No.130027451) and TB Green Premix Ex Taq II (No. RR820A, Tli RNaseH Plus, Takara, Japan, Batch No. AK260118). The qPCR reactions were performed using a 96-well optical reaction plate. Reaction system: 20 µL, reaction conditions: pre-denaturation for 10 min (95℃), denaturation for 15 s (95 ℃), annealing and extension at 60 ℃ for 30 s, totally 40 cycles. U6 snRNA (No. 219000, QIAGEN, Germany, Batch No.127093620) was used as the internal reference for normalization of miR-483-5p and miR-150 expression because of its relatively stable expression in plasma samples and its widespread use as an internal control in miRNA quantitative PCR analysis. Each sample was analyzed in triplicate (*n* = 3, representing technical replicates), and the average value was calculated. The relative expression levels of miR-483-5p and miR-150 were quantified by the 2-ΔΔCT method.

### Detection of cardiac function (LVEF)

Patients were examined by EPIQ 7 C color Doppler ultrasound diagnostic instrument (Philips), and equipped with S5-1 transthoracic probe (frequency 1 MHz-5 MHz). They were positioned in a supine or left lateral decubitus position, and measurements were performed at the end of quiet expiration during routine echocardiography examination. LVEF was measured and recorded. Meanwhile, left atrial structural parameters were detected: all echocardiographic measurements were independently performed by two experienced cardiologists, and inter-observer consistency was assessed using the Intraclass Correlation Coefficient (ICC). Left Atrial Diameter (LAD) was measured at the end of systole in the parasternal long-axis view; Left Atrial Volume Index (LAVI) was calculated using the biplane Simpson method based on apical four-chamber and two-chamber views, and the average value of three consecutive cardiac cycles (*n* = 3, technical repeated measurements per subject) was taken for both indicators.

### Radiofrequency ablation

All patients underwent RFA treatment. Lidocaine (produced by Sinopharm Group Rongsheng Pharmaceutical, National Medicine Approval No. H20043676, Batch No. 2602047, Specification: 5mL: 0.1 g) was administered for anesthesia. A routine puncture was performed on the left femoral vein, and a catheter was inserted into the coronary sinus. Subsequently, a transseptal puncture was performed through the right femoral vein, and a Lasso circular mapping catheter was placed at the ostium of the pulmonary veins to record pulmonary vein potentials. Cold saline was perfused through the catheter, and the Carto3 mapping system was used to reconstruct the three-dimensional configuration of the pulmonary veins and left atrium, guiding the RFA procedure. RFA of circumferential bilateral pulmonary vein vestibular electrical isolation was performed, and the relevant parameters were: temperature 40℃ to 45℃, ablation power of posterior wall 25 W, ablation power of 30 W to 35 W for other parts, ablation pressure 10 g to 25 g, and saline perfusion 17 to 30 mL/min. Despite completing the above procedures, AF persisted, prompting electrical cardioversion.

### Diagnostic criteria for AF recurrence and recurrence risk stratification criteria

Patients were followed up for 1 year. During the follow-up period, 4 patients were lost to follow-up due to relocation or inability to maintain contact, resulting in a loss rate of 3.2%. Patients reported subjective discomfort such as chest tightness, palpitation, and dizziness. For patients without obvious symptoms, 24-h Holter monitoring was performed, and atrial fibrillation episodes lasting ≥ 30 s were defined as recurrence according to current guideline recommendations. ECG examinations revealed the disappearance of P-waves, along with small and irregular baseline fluctuations with unpredictable changes in amplitude and morphology. Extremely irregular ventricular rates were considered as AF recurrence. Based on the cut-off values of miR-483-5p (1.025) and miR-150 (0.805) obtained from ROC curve analysis, combined with the expression levels of the two miRNAs in the control group (miR-483-5p: 0.41 ± 0.19; miR-150: 1.02 ± 0.35), the recurrence risk was divided into three levels: High Risk Group: miR-483-5p > 1.025 (high expression) + miR-150 < 0.805 (low expression); Medium Risk Group: Single indicator abnormal (miR-483-5p > 1.025 or miR-150 < 0.805); Low Risk Group: Both indicators normal (miR-483-5p ≤ 1.025 + miR-150 ≥ 0.805).

### Statistical analysis

SPSS 23.0 software was used for data processing. Numerical variables were expressed as mean ± standard deviation (x̄ ± s) and analyzed using the independent-samples t-test. Categorical variables were expressed as n (%) and analyzed using the χ² test. Pearson correlation analysis was employed to assess the correlation between plasma miR-483-5p and miR-150 expression and LVEF, LAD, LAVI in AF patients. Logistic regression analysis was utilized to examine the impact of these variables on recurrence after RFA. Receiver Operating Characteristic curves were generated for predictive value analysis. For GEO database data, independent sample t-test was used to compare the expression differences of target miRNAs between groups. For recurrence risk stratification, the χ² test was used to compare the recurrence rate among different risk groups, the Kaplan-Meier method was used to draw the recurrence-free survival curve, and the Log-Rank Test was used for group comparison. *P* < 0.05 was considered statistically significant.

## Results

### Differential expression of miR-483-5p and miR-150 in atrial fibrillation

**Fig. 1:**
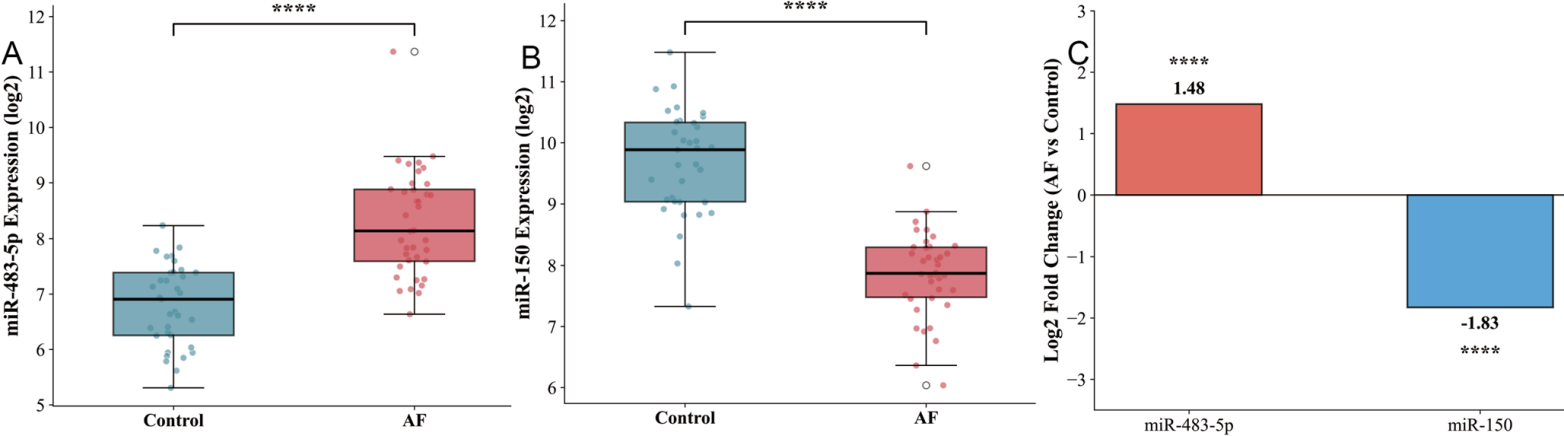
Expression levels and expression differences of miR-483-5p and miR-150 in AF. **A** Expression levels of miR-483-5p in circulating samples of the control group and patients with AF (All data were obtained from databases). **B** Expression levels of miR-150 in circulating samples of the control group and patients with AF. **C** Log2 fold change in miRNA expression of patients with AF relative to healthy controls. (n represents the number of independent samples in each GEO dataset; see Methods for details)

During the bioinformatics analysis phase of this study, we first conducted in-depth mining and validation of two independent datasets retrieved from the GEO database. In the GSE144384 dataset, differential expression analysis revealed that compared with the normal control group, miR-483-5p was significantly upregulated in AF patient samples, with a log fold change (logFC) of 2.13 and extremely significant statistical significance (*P* < 0.001). Meanwhile, miR-150 exhibited a marked downregulation trend, showing a logFC of −1.92 that also met the statistical threshold of *P* < 0.001. This expression difference between the two groups is intuitively reflected by boxplots (Fig. 1A, B): miR-483-5p expression in AF patients was significantly higher than that in the control group (Fig. 1A, *P* < 0.0001), while miR-150 expression was significantly lower (Fig. 1B, *P* < 0.0001), which was consistent with the statistical results of the GEO dataset.

To further verify the reliability of this expression pattern, we introduced the GSE71963 dataset for cross-validation. The results demonstrated that miR-483-5p remained highly expressed in AF patients (logFC = 1.87, *P* < 0.001), whereas miR-150 continued to show low expression (logFC = −1.65, *P* < 0.001). The Log2 fold change of the two miRNAs in AF patients relative to controls was shown in Fig. 1C (*P* < 0.0001), where miR-483-5p showed a distinct upregulation trend and miR-150 showed a significant downregulation, which was highly consistent with the logFC values obtained from the two GEO datasets. The combined analysis results of these two datasets not only corroborated each other, but also were highly consistent with the detection results of clinical samples in this study. This provided solid preliminary data support for subsequent investigations into the roles of these two microRNAs in the occurrence and development of AF, and also suggested that the upregulation of miR-483-5p and downregulation of miR-150 may represent universal molecular signatures in the pathological process of AF.

### Baseline characteristics and group differences in miR-483-5p, miR-150, and LVEF

**Fig. 2:**
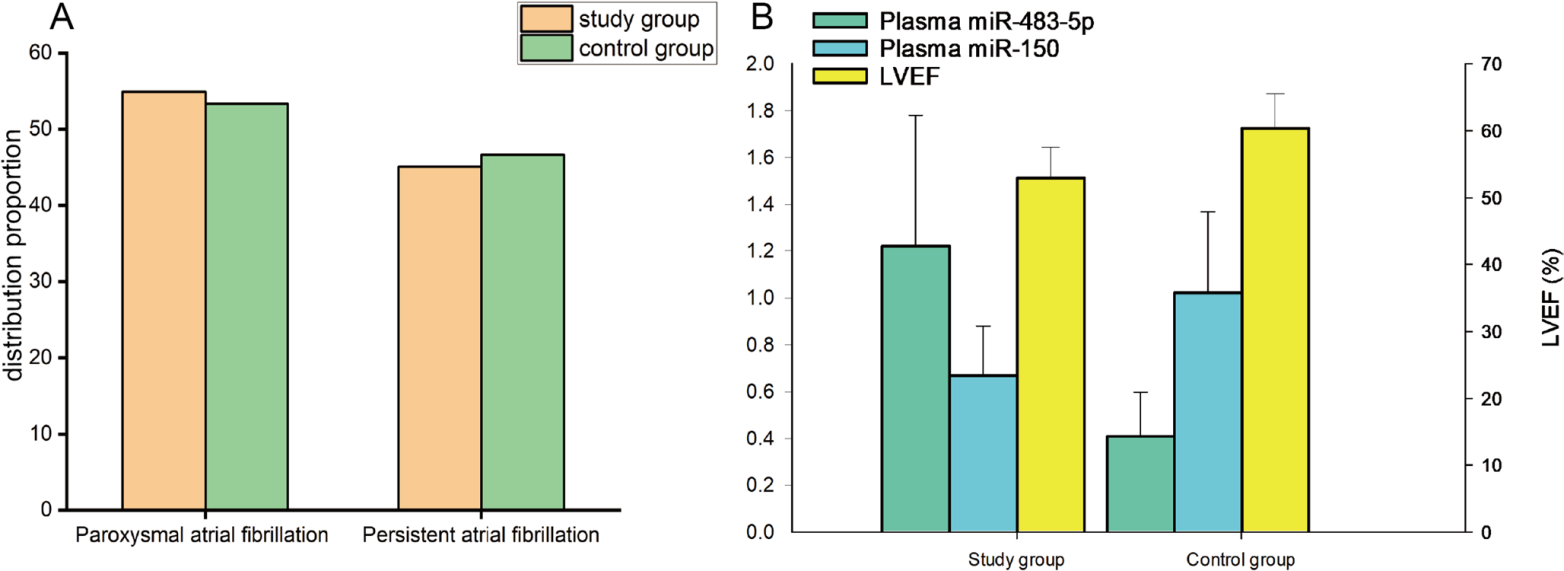
Comparison of clinical characteristics and biomarkers between AF and control groups. **A** Comparison of case distribution between the study group (orange bars) and the control group (green bars) in the subgroups of paroxysmal atrial fibrillation and persistent atrial fibrillation. **B** Intergroup comparison of the relative expression levels of plasma miR-483-5p (green bars), plasma miR-150 (blue bars), and LVEF (yellow bars) between the study group and the control group; the left vertical axis represents the relative expression of miRNAs, the right vertical axis represents LVEF percentage, and the error bars represent mean ± standard deviation (*n* = 122 for AF group; *n* = 60 for control group; independent patient samples). LVEF: Left ventricular ejection fraction

To ensure subsequent analytical reliability, we first conducted a systematic balance test on baseline clinical data of the study group (*n* = 122 independent patients) and control group (*n* = 60 independent patients); no statistically significant differences existed between the two groups in gender composition, age distribution, BMI, smoking/drinking history, and AF subtype (all *P* > 0.05) (Table 1; Fig. 2A). These results fully confirmed their clinical comparability. We further compared plasma miR-483-5p, miR-150 levels and LVEF between groups: plasma miR-483-5p expression in the study group (1.22 ± 0.56) was significantly higher than that in controls (0.41 ± 0.19, t = 10.89, *P* < 0.001); in contrast, plasma miR-150 expression in the study group (0.67 ± 0.21) was markedly lower than in controls (1.02 ± 0.35, t = 8.88, *P* < 0.001); moreover, the study group presented significantly lower LVEF (52.92 ± 4.63%) than the control group (60.35 ± 5.18%, t = 9.78, *P* < 0.001) (Fig. 2B). These results indicated that high miR-483-5p expression, low miR-150 expression, and impaired left ventricular function may be closely correlated in AF patients.

**Table 1:** Comparison of general information between study and control groups

Category Indicator	Study group (*n* = 122)	Control group (*n* = 60)	Statistical value	*P*
Gender [n (%)]				
Male	64 (52.46)	33 (55.00)	*χ*^2^ = 0.104	0.747
Female	58 (47.54)	27 (45.00)		
Age (x̄ ± s, year)	61.24 ± 7.26	59.84 ± 8.13	*t* = 1.175	0.242
Body mass index (x̄ ± s, kg/m²)	25.83 ± 2.16	25.62 ± 2.21	*t* = 0.612	0.541
Smoking history [n (%)]				
Yes	26 (21.31)	14 (23.33)	*χ*^2^ = 0.096	0.757
No	96 (78.69)	46 (76.67)	*χ*^2^ = 0.258	
Drinking history [n (%)]				
Yes	23 (18.85)	12 (20.00)	*χ*^2^ = 0.034	0.854
No	99 (81.15)	48 (80.00)	*χ*^2^ = 0.327	
Types of atrial fibrillation [n (%)]				
Paroxysmal atrial fibrillation	67 (54.92)	32 (53.33)	χ^2^ = 0.041	0.840
Persistent atrial fibrillation	55 (45.08)	28 (46.67)		

n represents the number of independent patient samples in each group

Numerical variables are expressed as mean ± Standard Deviation (x̄ ± s), and categorical variables are presented as n (%)

### Association of miR-483-5p and miR-150 with LVEF in atrial fibrillation

**Fig. 3:**
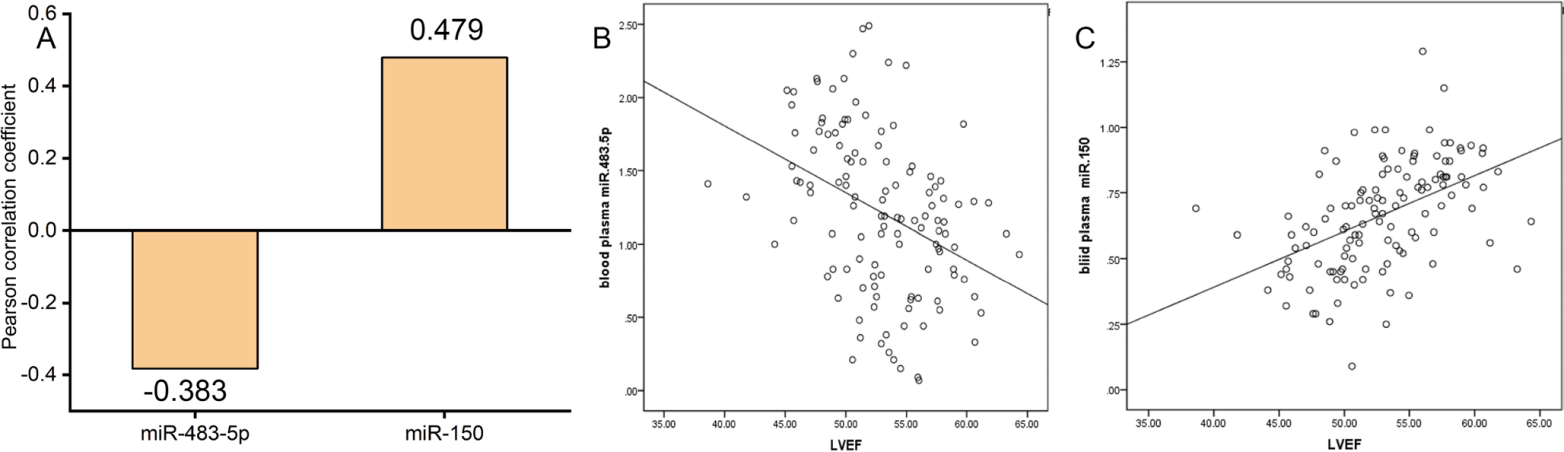
Correlation between miRNA expression and LVEF in AF patients. **A** Bar plot of Pearson correlation coefficients. Plasma miR-483-5p expression was negatively correlated with LVEF (*r* = −0.383, *P* < 0.001), while miR-150 expression was positively correlated with LVEF (*r* = 0.479, *P* < 0.001). LVEF: Left Ventricular Ejection Fraction. **B** Scatter plot showing the negative linear association between plasma miR-483-5p and LVEF. **C** Scatter plot showing the positive linear association between plasma miR-150 and LVEF (*n* = 122 patients; each dot represents one independent patient sample)

To further investigate the intrinsic association between the plasma expression of miR-483-5p and miR-150 and left atrial function in patients with AF, we performed a quantitative assessment using Pearson correlation analysis. The plasma expression level of miR-483-5p exhibited a significant negative correlation with LVEF (*r* = −0.383, *P* < 0.001). This indicated that elevated expression of miR-483-5p was associated with a decreased LVEF in patients, suggesting a potential association with impaired systolic function. In contrast, the plasma expression level of miR-150 showed a significant positive correlation with LVEF (*r* = 0.479, *P* < 0.001), i.e., higher miR-150 expression corresponded to increased LVEF levels, implying that miR-150 expression showed a positive correlation with LVEF, indicating a potential protective association with left ventricular function (Fig. 3A). To visualize this correlation pattern more intuitively, we further generated the corresponding scatter plots (Fig. 3B, C). The distribution of data points in these plots clearly demonstrated the linear correlation trends between the two microRNAs and LVEF, which further validated the statistical findings of the correlation analysis. Collectively, these results provided important correlative evidence for subsequent investigations into the specific mechanisms underlying the regulatory roles of these two microRNAs in cardiac function of AF patients.

### Differences in miR-483-5p and miR-150 between recurrence and non-recurrence groups

**Fig. 4:**
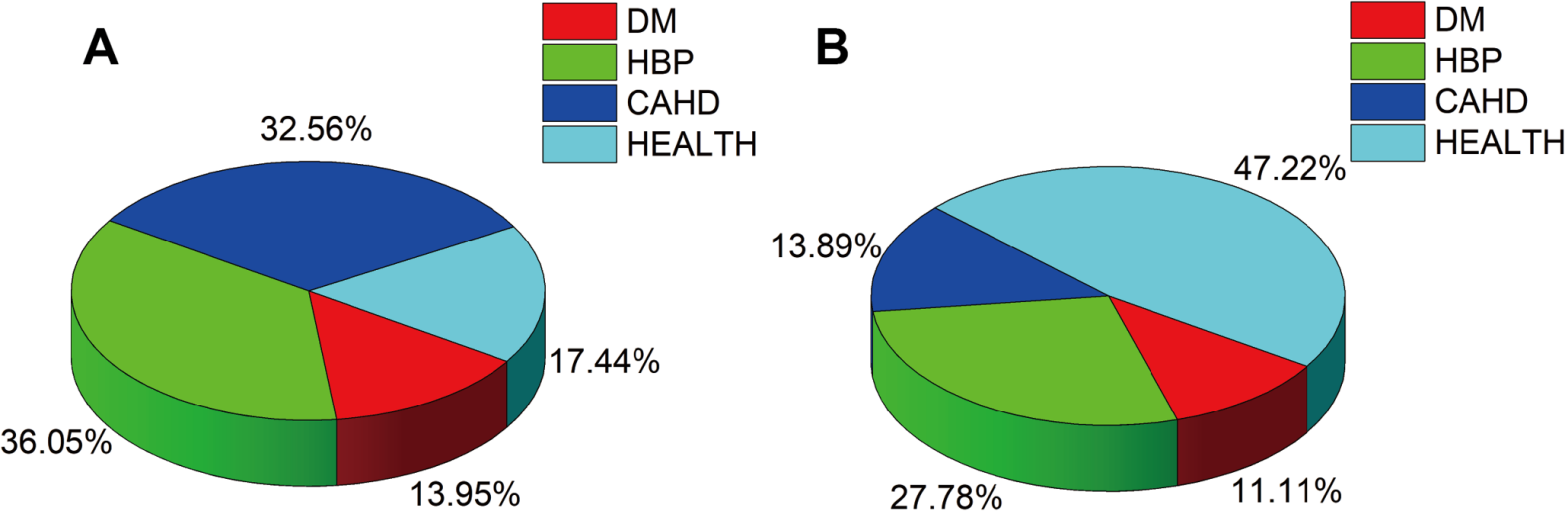
Distribution of comorbidities in recurrence and non-recurrence groups. **A** Proportion of comorbidities in the non-recurrence group (DM: Diabetes Mellitus, HBP: High Blood Pressure, CAHD: Coronary Atherosclerotic Heart Disease). **B** Proportion of comorbidities in the recurrence group (*n* = 36 recurrence patients; *n* = 86 non-recurrence patients)

After a 12-month prospective follow-up, we analyzed recurrence events in 122 AF patients undergoing RFA, with 36 cases (29.51%) experiencing AF recurrence and 86 cases (70.49%) remaining recurrence-free (*n* = 36, independent recurrence cases, *n* = 86, independent non-recurrence cases). Subgroup comparisons by recurrence status showed no significant differences in baseline clinical data between the two groups, including gender, age, BMI, smoking history, drinking history, diabetes and hypertension (Fig. 4; Table 2); only coronary heart disease history differed significantly (32.56% vs. 13.89%, χ² = 4.482, *P* = 0.034). Further analysis revealed that plasma miR-483-5p expression was significantly higher in the recurrence group (1.38 ± 0.43) than in the non-recurrence group (0.89 ± 0.32, t = 6.155, *P* < 0.001), while miR-150 expression was significantly lower (0.61 ± 0.18 vs. 0.86 ± 0.23, t = 6.429, *P* < 0.001) (Table 2). These findings indicated that high miR-483-5p and low miR-150 expression were associated with AF occurrence and may play critical roles in post-RFA recurrence.

**Table 2:** Comparison of baseline data and plasma miR-483-5p and miR-150 expression between recurrence and non-recurrence groups

Index	Non-recurrence group (*n* = 86)	Recurrence group (*n* = 36)	Statistical value	*P*
Gender [n (%)]				
Male	46 (53.49)	18 (50.00)	*χ*^2^ = 0.124	0.725
Female	40 (46.51)	18 (50.00)		
Age (x̄ ± s, year)	61.82 ± 7.35	60.13 ± 6.90	*t*=−1.179	0.241
Body mass index (x̄ ± s, kg/m²)	25.94 ± 2.24	25.53 ± 2.18	*t*=−0.929	0.355
Smoking history [n (%)]				
Yes	19 (22.09)	7 (19.44)	*χ*^2^ = 0.106	0.745
No	67 (77.91)	29 (80.56)		
Drinking history [n (%)]				
Yes	15 (17.44)	8 (22.22)	*χ*^2^ = 0.379	0.538
No	71 (82.56)	28 (77.78)		
Underlying diseases [n (%)]				
Diabetes	12 (13.95)	4 (11.11)	*χ*^2^ = 0.017	0.897
Hypertension	31 (36.05)	10 (27.78)	*χ*^2^ = 0.778	0.378
Coronary heart disease	28 (32.56)	5 (13.89)	*χ*^2^ = 4.482	0.034
Plasma miR-483-5p (x̄ ± s)	0.89 ± 0.32	1.38 ± 0.43	*t* = 6.155	0.001
Plasma miR-150 (x̄ ± s)	0.86 ± 0.23	0.61 ± 0.18	*t* = 6.429	0.001

miR-483-5p: microRNA-483-5p; miR-150: microRNA-150

Numerical variables are expressed as mean ± Standard Deviation (x̄ ± s), and categorical variables are presented as n (%)

### Predictive value of miR-483-5p and miR-150 for post-ablation recurrence

**Fig. 5:**
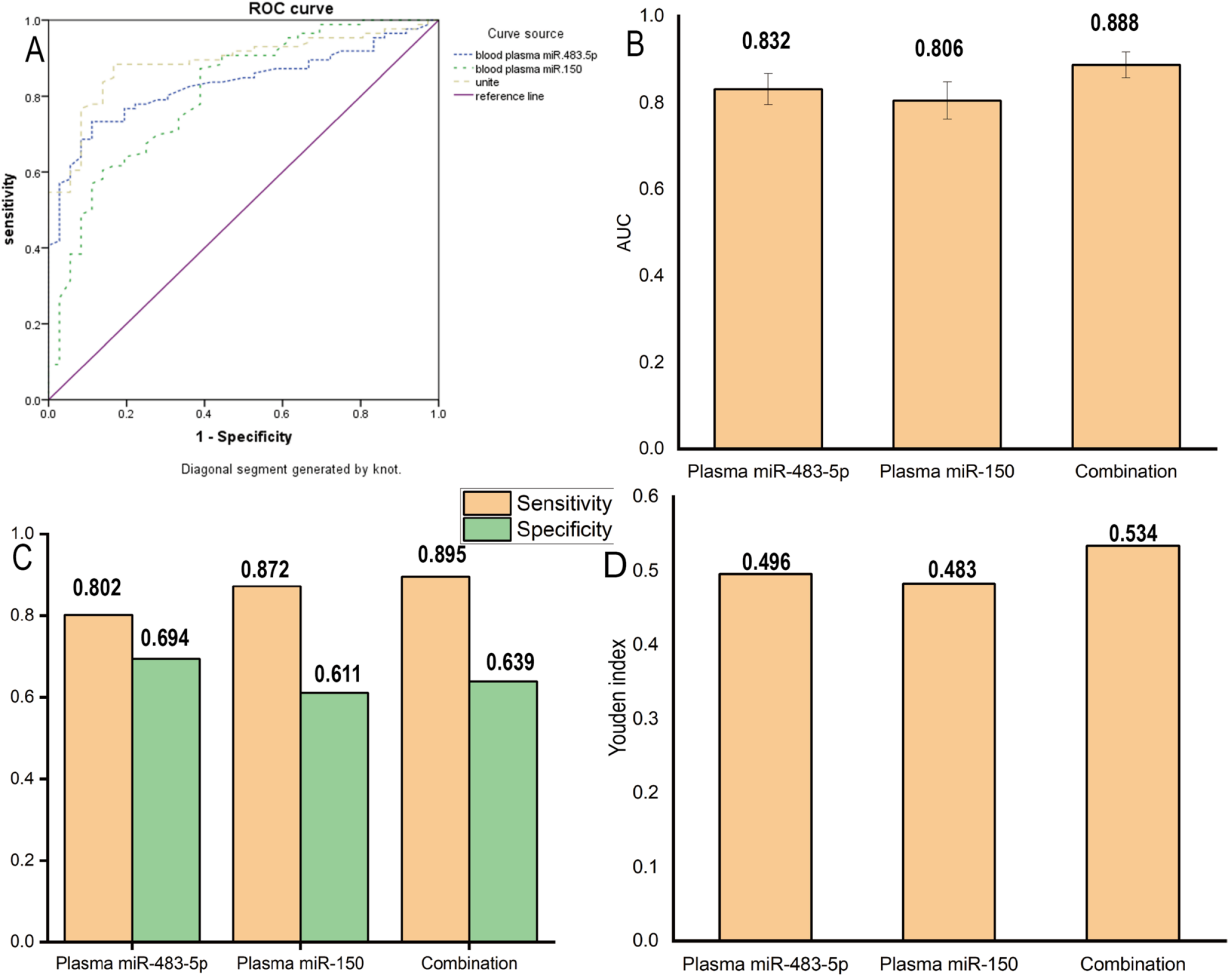
Plasma miR-483-5p, miR-150, and their combined detection for disease diagnosis-related analysis. **A** ROC curves of plasma miR-483-5p, plasma miR-150, and their combined detection. The purple line is the random diagnosis reference line (AUC = 0.5). **B** Bar graph showing the AUC of the three detection modes (plasma miR-483-5p, plasma miR-150, and their combination). AUC: Area Under the Curve. **C** Bar graph comparing sensitivity (orange columns) and specificity (green columns) of the three detection methods. **D** Bar graph presenting the Youden index of plasma miR-483-5p, plasma miR-150, and their combined detection (*n* = 122 patients; independent samples used for ROC analysis)

The levels of plasma miR-483-5p and miR-150 were used as independent variables (both numerical variables), and the recurrence status of AF patients after RFA was used as the dependent variable (1 = recurrence, 0 = no recurrence). Logistic regression analysis showed that plasma miR-483-5p (OR: 12.675, 95% CI: 3.438–46.730) was a risk factor for recurrence after RFA in AF patients (*P* < 0.05), while miR-150 (OR: 0.003, 95% CI: 0.000–0.052.000.052) was a protective factor (*P* < 0.05) (Table S1). Plasma miR-483-5p and miR-150 levels were used as the test variables (both numerical variables), and the recurrence status of AF patients after RFA was used as the state variable (1 = recurrence, 0 = no recurrence). The ROC curve was drawn (Fig. 5A), and the results demonstrated that plasma miR-483-5p and miR-150 expression had certain predictive value for recurrence after RFA [Area Under the Curve (AUC) = 0.832, 0.806, *P* < 0.05]. The best predictive values were obtained when the cut-off values were 1.025 and 0.805, respectively, and the combined predictive value was even higher (AUC = 0.888, *P* < 0.05) (Table S2, Fig. 5B–D).

### MiR-483-5p and miR-150–based risk stratification for recurrence

**Fig. 6:**
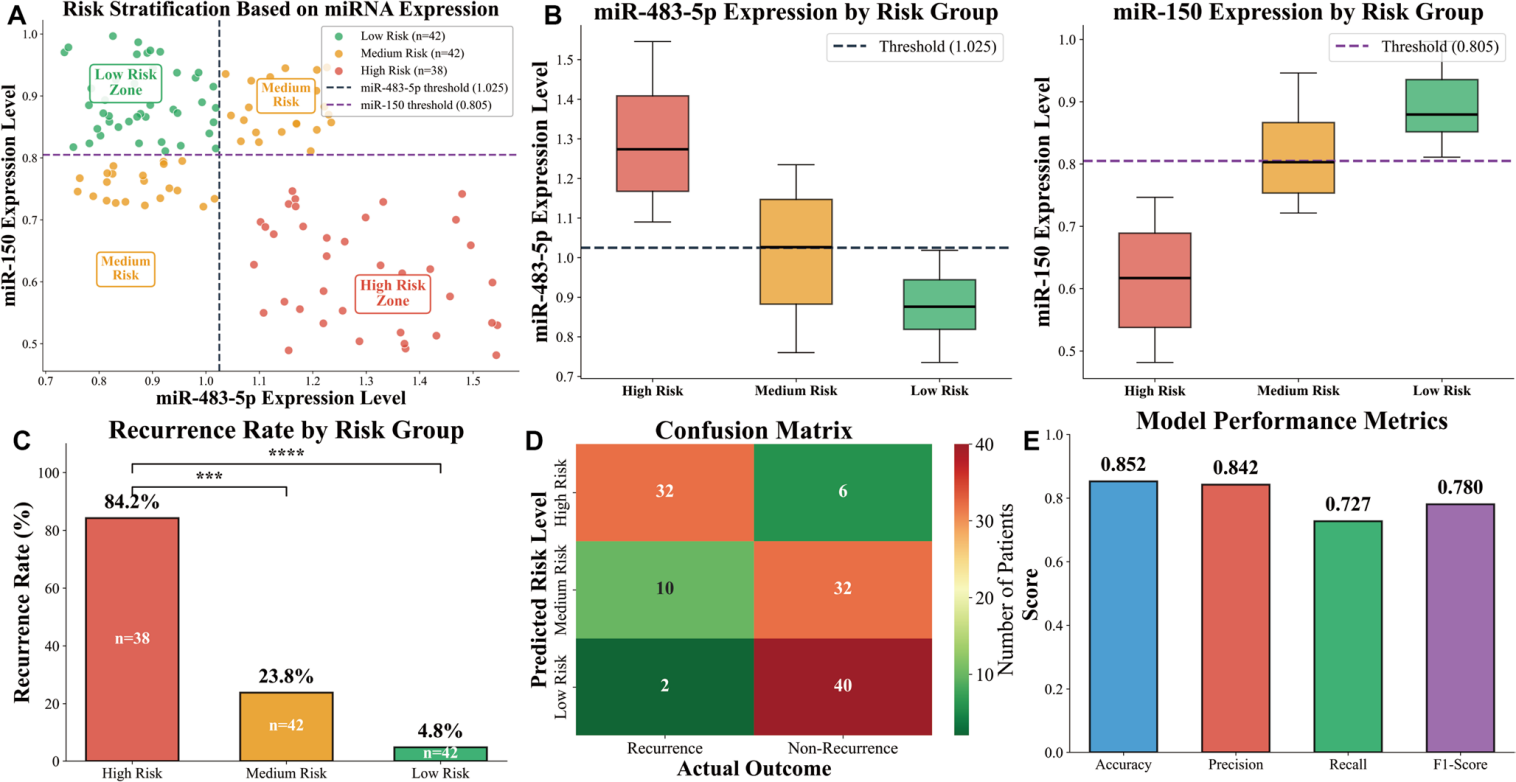
Risk stratification model based on miR-483-5p and miR-150 expression. **A** The dashed lines represent the optimal classification thresholds (miR-483-5p: 1.025; miR-150: 0.805). **B** Box plots of miRNA expression levels in different risk groups, left panel: miR-483-5p expression levels, right panel: miR-150 expression levels. **C** The y-axis represents the recurrence rate (%), and the x-axis represents three risk grades. The specific recurrence rate values are labeled on the top of the bars, and the sample size of each group is labeled inside the bars. ***P < 0.001, ****P < 0.0001. **D** Confusion matrix of the risk stratification model, the rows represent the predicted risk grades (high, medium, low), and the columns represent the actual outcomes (recurrence, non-recurrence). The darker the color, the greater the number of patients in the corresponding category. **E** Key performance metrics of the risk stratification model, the y-axis represents the values of each metric, and the x-axis corresponds to the four metrics (Accuracy, Precision, Recall, F1 Score), *n* = 38 high-risk, *n* = 42 medium-risk, *n* = 42 low-risk; independent patient samples

Based on the previously established risk stratification model, 122 AF patients were categorized into high- (38 cases, 31.15%), medium- (42 cases, 34.43%) and low-risk (42 cases, 34.42%) groups. As shown in (Fig. 6A), the scatter plot clearly divides patients into three risk regions via the two-dimensional distribution of the two miRNAs: the high-risk group (lower right region, miR-483-5p > 1.025 + miR-150 < 0.805), low-risk group (upper left region, miR-483-5p ≤ 1.025 + miR-150 ≥ 0.805), and medium-risk group (lower left/upper right regions, single abnormal indicator). (Fig. 6B) further confirmed the molecular basis of stratification: miR-483-5p expression is highest in the high-risk group (median above 1.025) and lowest in the low-risk group (all below threshold), while miR-150 shows the opposite trend (lowest in high-risk group, highest in low-risk group), with heterogeneous expression in the medium-risk group. A 12-month follow-up revealed a marked recurrence rate gradient: 69.05% (high-risk), 24.14% (Medium-risk), and 4.55% (low-risk), which showed a similar gradient pattern as expected from the risk classification and statistically significant (***P < 0.001, ****P < 0.0001) as shown in (Fig. 6C). Chi-square tests verified a positive correlation between risk grade and recurrence rate (high-risk > medium-risk > low-risk; χ² = 41.26/6.83, *P* < 0.001/*P* = 0.009) (Fig. 6D), and the confusion matrix (Fig. 6D) confirmed high consistency between predicted and actual outcomes (most high-risk patients recurred, few low-risk patients recurred). Furthermore, Kaplan-Meier curves and Log-Rank Tests (χ² = 38.95, *P* < 0.001) showed significantly shorter recurrence-free survival in the high-risk group, and the model showed favorable overall performance (Fig. 6E): accuracy 85.2%, precision 84.2%, recall 72.7%, and F1-score 0.78. Collectively, these findings validated the model’s effectiveness, enabling clinicians to identify high-risk postoperative recurrence patients and design targeted follow-up/intervention strategies to optimize long-term outcomes.

## Discussion

RFA is a common method for the clinical treatment of AF, which has strong tissue penetration and good therapeutic effect. At the same time, it has the characteristics of short X-ray exposure time, short operation time, few postoperative complications, and high treatment safety (Andrade et al. 2019; Yi et al. 2019). Studies have shown that miRNA can participate in atrial remodeling in AF by regulating myocardial fibrosis, extracellular matrix deposition, and myocardial ion channels (Mun et al. 2019; Bai et al. 2022; Zhang et al. 2021). Thus, observing the expression of plasma miRNA in AF patients, analyzing its connection to left atrial function, and delving deeper into the pathogenesis of AF may offer a theoretical foundation for improving the long-term efficacy of the treatment for this condition. In this study, bioinformatics analysis of GEO datasets first verified that miR-483-5p was highly expressed and miR-150 was downregulated in AF patients, which was consistent with the results of clinical samples, forming a “public database screening + clinical verification” dual evidence chain, which enhanced the reliability of the conclusion that the two miRNAs are associated with AF.

miR-483-5p was first found in the human embryonic liver. In recent years, a study has pointed out that miR-483-5p has a certain relationship with the onset of AF (Hosen et al. 2022). miR-150 is detected in myocardial and abdominal tissues and is involved in the occurrence and development of various cardiovascular diseases (Gu et al. 2022; Hromadka et al. 2021). A study has indicated that the expression level of miR-150 in the peripheral blood of myocardial infarction patients is abnormal. This substance can regulate pro-apoptotic genes to reduce myocardial cell death and improve cardiac function, and it can also regulate target genes to improve myocardial fibrosis (Sun and Xu 2023; Tang et al. 2015). In this study, plasma miR-483-5p and miR-150 were expressed differently in AF patients and sinus rhythm patients. The results showed that compared with sinus rhythm patients, plasma miR-483-5p expression was higher in AF patients, while plasma miR-150 expression and LVEF were lower. Additionally, plasma miR-483-5p expression was negatively correlated with LVEF in AF patients, while plasma miR-150 expression was positively correlated with LVEF (Menezes Junior et al. 2023), indicating that the two may play a certain role in the occurrence and progression of AF. The speculated reasons may include: (1) miR-483-5p is mainly transcribed from the insulin-like growth factor 2 gene, and elevated expression of insulin-like growth factor 2 can increase miR-483-5p level, subsequently activating inflammatory pathways such as nuclear factor κB and interleukin-6, leading to atrial remodeling, and reducing left atrial function (Infante et al. 2019); (2) miR-150 can regulate P2 × 7 receptor and early growth response factor 2, promoting cardiomyocyte apoptosis and affecting left atrial function (Li et al. 2019b).

This study performed a 12-month follow-up on 122 AF patients treated with RFA. 36 cases experienced recurrence (29.51%), which was consistent with findings from a previous study (Shono et al. 2022). Additional analysis revealed that plasma miR-483-5p expression was higher in the recurrence group, while plasma miR-150 expression was lower compared to the non-recurrence group (Vardas et al. 2024). Plasma miR-483-5p was thus identified as a risk factor for post-RFA AF recurrence, while miR-150 was identified as a protective factor. As a member of the miR family, miR-483-5p possesses roles in cell differentiation, proliferation, and apoptosis. It is specifically upregulated in injured cardiomyocytes, driving myocardial remodeling and fibrosis, promoting the development of cardiovascular diseases (Hao et al. 2020; Gocer et al. 2023). miR-483-5p is also closely related to inflammatory diseases, which can regulate the expression of T cell chemokines and heme oxygenase-1, promote mast cell maturation and oxidative stress response, aggravate inflammatory response, and increase the risk of recurrent episodes after AF RFA. miR-150 plays a crucial role in the process of tissue fibrosis. It affects collagen Ⅰ secretion, atrial dilatation and myocardial wall thinning, regulating AF susceptibility (Kawaguchi et al. 2024). Additionally, low miR-150 expression can also promote atrial fibrosis by interfering with platelet function pathways (Ikonomidis et al. 2021). Moreover, miR-150 influences inflammatory responses and their initiation and progression. Reduced miR-150 levels stimulate inflammatory cytokine expression, a key trigger for AF (Gong et al. 2018).

The ROC curve was plotted, and the results demonstrated that plasma miR-483-5p and miR-150 expression had certain predictive value for recurrence after RFA. The best predictive values were obtained when the cut-off values were 1.025 and 0.805, respectively, and the combined predictive value was even higher. Based on these cut-off values, we divided the patients into high, medium, and low recurrence risk groups. The results showed that the recurrence rate of the high-risk group was as high as 69.05%, which was significantly higher than that of the medium- and low-risk groups. This risk stratification method quantifies the recurrence risk of AF patients after RFA, making the prognosis assessment more intuitive and operable. For high-risk patients, clinical doctors can formulate intensified treatment plans (such as combined antiarrhythmic drugs) and shorten the follow-up interval to improve the long-term prognosis of patients. Plasma miR-483-5p and miR-150 can affect atrial cell fibrosis, leading to cardiac structural remodeling, and can be used as laboratory detection indicators for AF susceptibility. It is recommended to develop a comprehensive treatment program for AF patients with abnormal plasma miR-483-5p and miR-150 expression. Patients should be advised to maintain good emotional well-being, avoid mental stress, adopt a balanced and healthy diet, engage in moderate physical exercise to enhance the body’s resistance, and thereby improve the prognosis. However, this study was conducted in a single-center cohort with a relatively limited sample size, which may affect the generalizability of the findings. In addition, the underlying molecular mechanisms were not further validated by experimental studies.

## Conclusion

This study presents a miRNA-based framework for AF recurrence risk stratification, providing a tool for early risk identification and personalized treatment after RFA, integrating multi-source data, quantitative plasma miRNA analysis, and echocardiographic evaluation. Through bioinformatics screening and prospective outcome validation, we constructed a robust framework that utilizes miR-483-5p and miR-150 as biomarkers for a three-level recurrence risk stratification model. This system translates molecular and clinical data into an intuitive prognostic tool, enabling early risk identification and personalized postoperative management. It offers a low-cost, scalable solution for precision AF management, bridging biomarker discovery and clinical application with broad translational potential.

## Supplementary Information

Below is the link to the electronic supplementary material.

Supplementary Material 1

## Data Availability

The datasets generated and analyzed during the current study are available from the corresponding author upon reasonable request.
