# Supplementary material for: A risk model based on miR-483-5p and miR-150 for atrial fibrillation recurrence
Source: 3 Biotech. 2026 May 20;16(6):217. doi: 10.1007/s13205-026-04842-8 (PMC13190974; doi:10.1007/s13205-026-04842-8)
Supplement: Supplementary file 1 — Supplementary Material 1 [file 13205_2026_4842_MOESM1_ESM.docx]

## Supplementary informations

**Table S1.** Effect of plasma miR-483-5p and miR-150 expression on recurrence after RFA

| **Index** | ***β*** | **Standard error** | ***Wald χ*^2^** | ***P*** | ***OR*** | **95%*CI*** | |
| --- | --- | --- | --- | --- | --- | --- | --- |
|  |  |  |  |  |  | **Lower limit** | **Upper limit** |
| **Plasma miR-483-5p** | 2.540 | 0.666 | 14.554 | 0.000 | 12.675 | 3.438 | 46.730 |
| **Plasma miR-150** | -5.704 | 1.397 | 16.662 | 0.000 | 0.003 | 0.000 | 0.052 |
| **Constant quantity** | 2.126 | 1.280 | 2.761 | 0.097 | 8.381 | - | - |

Note: miR-483-5p: microRNA-483-5p; miR-150: microRNA-150; RFA: radiofrequency ablation.

**Table S2.** Predictive value of plasma miR-483-5p and miR-150 expression for recurrence after RFA

| **Test variable** | **AUC** | **Standard error** | ***P*** | **95%*CI*** | | **Cut-off value** | **Sensitivity** | **Specificity** | **Youden index** |
| --- | --- | --- | --- | --- | --- | --- | --- | --- | --- |
|  |  |  |  | **Lower limit** | **Upper limit** |  |  |  |  |
| **Plasma miR-483-5p** | 0.832 | 0.036 | 0.000 | 0.761 | 0.903 | 1.025 | 0.802 | 0.694 | 0.496 |
| **Plasma miR-150** | 0.806 | 0.043 | 0.000 | 0.721 | 0.892 | 0.805 | 0.872 | 0.611 | 0.483 |
| **Combination** | 0.888 | 0.030 | 0.000 | 0.829 | 0.947 | - | 0.895 | 0.639 | 0.534 |

Note: miR-483-5p: microRNA-483-5p; miR-150: microRNA-150; RFA: radiofrequency ablation; AUC: area under the curve.
